# Supplementary material for: Are carbon emissions trading and green financial instruments synergistic? -Comprehensive quantitative research based on content analysis
Source: PLoS One. 2024 Mar 7;19(3):e0298601. doi: 10.1371/journal.pone.0298601 (PMC10919601; doi:10.1371/journal.pone.0298601)
Supplement: S1 Data — (ZIP) [file pone.0298601.s001.zip › Data/地级市Z_F工作报告.zip!/╣π╢1⁄2/╒┐╜¡╩╨/└┤╫╘╣1⁄2╓┌║┼í╛┬φ┐╦╩2╛▌═°í┐_2019.docx]

各位代表：

　　现在，我代表市人民政府向大会作政府工作报告，请予审议，并请政协委员和其他列席人员提出意见。

　　2018年工作回顾

　　过去一年，是中国改革开放40周年，是全面贯彻党的十九大精神的开局之年，也是湛江富有开创意义的一年。一年来，我们在市委坚强领导下，在市人大、市政协监督支持下，坚持以习近平新时代中国特色社会主义思想为指导，全面贯彻党的十九大和十九届二中、三中全会精神，深入贯彻落实习近平总书记对广东重要讲话精神，统筹推进“五位一体”总体布局，协调推进“四个全面”战略布局，坚持稳中求进工作总基调，奋力从更高层次更大格局谋划中贯彻落实新发展理念，做好“四篇文章”；在聚力推进“四大抓手”“五大产业发展计划”和全面推进经济社会及各项事业振兴发展中努力推动高质量发展。我们实现了“稳、快、好”的发展预期，呈现了新的发展气象。

　　2018，贯彻新发展理念，湛江拥有了新定位。这一年，习近平总书记多次提及湛江，充分肯定湛江作为首批沿海开放城市在国家对外开放格局中的重要地位和作用，要求“把汕头、湛江作为重要发展极，串珠成链，打造现代化沿海经济带”，要求湛江加强与海南自贸区对接合作、相向而行，省委要求湛江建设省域副中心城市，湛江发展定位被提升到前所未有的战略新高度。

　　2018，贯彻新发展理念，湛江筑建了新基础。这一年，湛江铁路枢纽总图编制完成，五条高铁以湛江为中心无缝对接融入国家高铁网。深湛高铁江湛段开通，湛江历史性迈入“高铁时代”。湛江港即将成为华南地区唯一通航40万吨级船舶的世界级深水港口。全年货物吞吐量突破3亿吨，稳居全省第2位、北部湾地区首位。

　　2018，贯彻新发展理念，湛江构建了新格局。这一年，我们高起点编制新时代新一轮城市总体规划，强化产业支撑，力促海西提质，推进海东开发，坚持“双轮驱动”推动县域壮大，落实“七大行动”推进乡村振兴，宜居宜业宜游生态型海湾城市格局逐步彰显。德国巴斯夫集团投资100亿美元的精细化工一体化项目落地签约，成为新时代中国对外开放两大标志性项目之一，受到党和国家领导人高度赞赏，与湛江钢铁、中科炼化等重大产业项目构建起湛江先进制造业主体框架。举办2018湛江（深圳）投资合作推介会等一系列商贸活动，签约130个项目，总投资2159亿元，一大批国内外500强企业进驻落户。现代农业、现代服务业、军民融合等产业蓬勃发展，具有湛江特色的现代化产业格局初步形成。

　　2018，贯彻新发展理念，湛江转换了新动能。这一年，我们发挥连接粤港澳大湾区、海南自贸区、北部湾城市群三大国家战略节点优势，谋划建设国家现代化沿海经济带雷州半岛先行区，探索湛江深水港与粤港澳大湾区港口合作。湛江入选全国最具投资潜力城市50强。中国海博会国际化、专业化水平大幅提升，聚焦展示了湛江开放发展的豪情与自信。国家高新区、国家海洋经济发展示范区、广东省实验室等高规格战略平台密集落户，湛江创新发展有了更为强劲的载体支撑。

　　2018，贯彻新发展理念，湛江创造了新质量。这一年，我们坚定走高质量效益型发展之路，推动经济社会发展提质、增效。全市生产总值突破3000亿元，预计达到3012亿元，增长6%，实现历史性跨越。实现规上工业增加值770亿元，增长5.2%。工业企业经济效益综合指数排名全省第2。完成固定资产投资1260亿元，增长12.8%。实现社会消费品零售总额1690亿元，增长10.2%。完成外贸进出口总额377亿元，增长9%。居民人均可支配收入21170元，增长8%。来源于湛江财政总收入678.3亿元，增长16.2%。一般公共预算收入122亿元，完成年度预算的103.8%，按可比口径同比增长15.4%，其中税收收入90.3亿元，增长19.6%，增速排名全省第3，非税收入占比从44.1%大幅下降到25.9%，收入质量历年最优。

　　一年来，全市上下团结一致，奋力拼搏，主要抓了以下工作。

　　（一）全力推进交通大会战，现代化立体交通脉络成型

　　坚持着眼长远、科学规划、批次推进、落地见效，坚持“海陆空铁”齐头并进，全年完成投资166.6亿元，增长61%，增速创历史新高。湛江国际机场预可研报告获国务院批准，航站楼动工建设，同步规划配套铁路和空港经济区。广湛客专可研报告通过评审，张海高铁完成初步方案研究。高铁西站投入使用。东海岛铁路湛江西至钢厂段建成通车。湛徐高速徐闻港支线、徐闻港进港公路改扩建工程建成；广东滨海旅游公路湛江段完成工可报告编制；调顺跨海大桥主桥设计方案完成优化并全面动工；环城高速南三岛大桥具备开工条件，茂湛高速改扩建工程开工；玉湛高速、东雷高速、汕湛高速吴川支线加快建设。湛江港40万吨级航道进入施工、监理招标阶段，全面开工在即；霞山港区通用码头工程动工、散货码头竣工验收，东海岛港区杂货码头、中科炼化项目配套码头、徐闻港区南山客货滚装码头主体工程加快建设，大唐雷州电厂配套码头建成。

　　（二）全力推进产业转型升级，实体经济质量效益凸显

　　重大工业项目有序推进。重点项目完成投资420亿元，其中省重点项目完成投资380亿元，完成年度计划的118.3%，完成投资额居粤东西北地区第一。钢铁、石化、造纸三大产业实现工业产值1100亿元，占全市规上工业总产值“半壁江山”。湛江钢铁基地成为中国内地唯一获“菲迪克奖”的产业项目，全年生产钢材790万吨，实现产值381亿元，投资188.5亿元的3号高炉系统项目启动建设。中科炼化项目完成投资100亿元，累计完成投资180亿元，完成了总投资的52％。湛江晨鸣产销两旺，实现产值136亿元，创历史新高。大唐雷州电厂完成投资22亿元，累计完成投资69亿元，完成总投资的75％。9项重点输变电工程建成投产。实施工业企业技术改造三年行动计划，新增规上工业企业120家。廉江成为省小家电智能制造区域创新中心，实现我市省级制造业创新中心零的突破。

　　产业园区持续扩能增效。完成园区规上工业总产值1989亿元、实现增加值568亿元，分别增长12%、13%；实现税收200亿元，增长12.8%；承接珠三角转移项目18个，新落地项目34个，完成投资25.3亿元。霞山临港、湛江、廉江产转园获省资金和用地奖励，海东、遂溪、奋勇产业集聚地升级为省级产业园。全市园区新增面积2.2万亩，增长15.4%，省级产业园达到13个，成为全省拥有省级园区最多的地级市。

　　现代服务业提质发展。大力构建电子商务、全域旅游、信息与金融服务等现代服务业多元业态。信息服务业提速发展。与顺丰、阿里巴巴分别开展水产配送和农村电商合作。湛江港集团成为全国供应链创新应用试点企业。遂溪、廉江成为省电子商务进农村综合示范县（市）。发布全国首个海洋科技大数据综合平台——“海上云”。电信业务总值达到244.6亿元，增长1.1倍。现代旅游蓬勃发展。引进12个亿元级旅游项目，华侨城集团计划500亿元投资湛江滨海旅游，湛江成为华侨城开发全域旅游全国首个地级市；携手招商局集团打造“北部湾蛇口”，总投资75亿元的国际邮轮港综合体项目正式启动；引进华强方特“东方神画”主题乐园项目。德萨斯水世界乐园在鼎龙湾国际海洋生态度假区正式营业。成功举办深湛高铁旅游发展大会，湛江高铁旅游市场初步形成。机场旅客吞吐量突破250万人次，增长20％。接待游客5172万人次、增长18.9%，旅游收入511.2亿元、增长21.3%。现代金融加快发展。新增金融机构4家，总数达127家。南粤银行跃升全球银行500强。本外币存款余额3343.6亿元，贷款余额2164.2亿元，存贷比64.7%；实现金融业增加值94亿元。

　　创新驱动发展成效明显。在全省率先启动军民融合深度发展战略规划编制，中船重工集团首家集研发产业一体化企业注册，中船重工国家重大科技专项保障基地落户。湛江海洋科技产业创新中心启动运营。国家知识产权示范城市进入培育阶段，专利申请5794件、增长20.6%，专利授权4441件、增长71.8%，专利申请和授权量均创历史新高。深入实施人才战略，引进省“扬帆计划”技术团队，新增两家省企业重点实验室。我市科技工作者李中荣获素有“中国诺贝尔奖”之誉的“何梁何利基金奖”。支持172家规上企业开展技改，5家规上工业企业实现“机器换人”。新增高新技术企业74家，总数达198家，增长58.4%，高新技术产业总产值突破700亿元、增长15%。

　　（三）全力深化改革开放，发展活力和内生动力持续增强

　　全面改革纵深推进。抓重点带全局，抓关键促突破，协同推进各领域改革。市县两级政府机构改革全面实施。“放管服”改革持续深化，清理规范中介服务事项38项，取消调整行政职权27项；推进“减证便民”行动，取消66项市政府部门证明事项；推行企业“二十四证合一”“一口办理”；企业开办时间缩减到5个工作日。推广全程电子化登记，商事登记事项全部实现网上办理。率先在粤东西北地区启动“中介服务超市”，进驻147项财政性资金项目。国资国企改革取得新成效，完成72家国有“僵尸企业”出清重组；实施湛化集团环保搬迁和市污水公司增资扩股，市基投集团、交投集团分别成功发行10亿元债券。国税地税征管体制改革落地，盐业体制改革全面完成。推进中小学教师“县管校聘”管理改革；完成农垦基础教育移交地方政府管理工作。公立医院开展薪酬改革试点，取消医用耗材加成，医改考评成绩位居全省第3。

　　市场活力持续增强。落实减税降费政策，降低企业税费成本48.2亿元，减免涉企行政事业性收费5.7亿元，降低实体经济企业综合成本65亿元。新增市场主体5万户、增长22.7%，新设外商投资企业143家、增长6.5倍；新增“个转企”3554户，增长19.3倍。税收超千万元民营企业达30家，恒兴水产入选省民营企业100强。民间投资超740亿元、增长10%，民营经济增加值超1900亿元、增长6.5%，创造了60%以上的GDP和固定资产投资、70%以上的创新成果和税收，提供了80%以上的市场主体和城镇就业岗位。

　　开放发展呈现新面貌。与“一带一路”沿线国家贸易总额86亿元，市场采购出口20.6亿元。新增1个国际友城。成功举办中国海博会，72个国家、2832家企业参展，达成合作交易意向1100亿元。湛江海关机构改革强力推进，新设和升级5个正处级隶属海关。跨境贸易便利化水平不断提升，整体通关时间压缩1/3，湛江港口岸核心能力复核获得全国海港评分第一。积极融入粤港澳大湾区建设，与深圳南山区签订高新技术产业园合作框架协议。坚持与海南相向而行，与北部湾10个地级市在交通、农业等多领域达成合作协议。与黑龙江省绥化市达成产业合作项目10个、合作金额31亿元。

　　（四）全力推进区域协调发展，城乡融合共进呈现新格局

　　中心城区强芯提质效果明显。开展湛江干线路网、市区轨道交通规划编制。首条城市快速主干道——湛江大道动工建设。西客站综合交通枢纽工程建成使用。疏港大道隧道工程基本完成。西城快线等22条市政道路建成通车。完成赤坎立交桥危桥拆除和临时道路建设。完成39条小街小巷升级改造。建成综合交通大数据中心。城市更新步伐加快，完成“三旧”改造面积2050亩；引入著名央企华润置地高水平推进旧大天然片区、南油片区组团式升级改造。启动实施湛江市引调水工程。抓好“创文”“巩卫”工作，持续推进城市“六乱”“两违”整治，完成年度省定违建治理目标。高新区海东园区开展规划编制，14个重点项目动工。

　　乡村振兴战略深入实施。实现农业总产值788亿元，增长5.5％。粮食总产量146.76万吨，增长3.8%，粮食安全责任考核获省优秀。新增农业部水产健康养殖示范场4个，创建“徐闻菠萝”农产品地理标志认证，新增省重点农业龙头企业7家、省级现代农业产业园6个，遂溪县火龙果产业园经验在全省推广。农村土地确权工作基本完成，农村集体产权制度改革全面铺开。4个小镇纳入第二批省特色小镇培育库。1812条贫困自然村完成“三清三拆”村容村貌整治，1825条贫困自然村设置垃圾收集点，624条自然村建成终端污水处理设施。“四好农村路”试点县（镇）创建工作扎实推进，硬化自然村村道124.4公里，改造乡道113.2公里。96宗水利项目开工建设；完成重点中型灌区节水改造工程3宗，恢复和新增有效灌溉面积14万亩。实施垦造水田4.6万亩，垦造水田“雷州模式”在全省推广。新一轮农网改造完成投资9.3亿元，省定贫困村电网改造全面完成。

　　（五）全力打好“三大攻坚战”，高质量发展基础更加坚实

　　有效防范化解重大风险。全力防范化解地方政府隐性债务，实行地方政府债务限额管理，整体债务规模控制在省下达的债务限额内。盘活财政资金资产，新增债券资金51.9亿元。加快市属投融资平台市场化转型，规范PPP项目操作。压实金融风险防控属地管理责任，深入开展金融风险排查和互联网金融风险专项整治。9家农信社改制取得突破，清收压降不良贷款21.86亿元，雷州、遂溪农商行挂牌营业。

　　脱贫攻坚扎实有效。投入财政扶贫资金24.7亿元，推动5.5万名贫困群众预脱贫。实施产业帮扶项目15.1万个，在全省率先开展贫困户信用等级评定及奖补，激励贫困群众自力更生、争先脱贫。开展贫困户技能培训1.5万人次，转移就业5.6万人次。完成贫困户危房改造4959户。与柳州扶贫协作成效明显，带动150条贫困村、8.7万贫困人口预脱贫。

　　污染防治扎实开展。全面实行“河长制”，在全省率先推行“湾长制”，8个考核断面水质达标，鹤地水库渠首断面水质达到三类。开展江河湖库“五清”行动，水环境质量总体保持优良。推动工业污染源、面源污染和机动车污染防治，超额完成省下达的污染减排任务，环境空气质量综合指数全省第2。布设土壤质量国控点位102个、省控点位180个，土壤环境质量监测网络基本成型。落实海洋、自然资源和环保督察整改。省环保考核获得优秀。推进新一轮绿化大行动和雷州半岛生态修复，完成造林更新6.5万亩，新建（改造）森林和湿地公园5个，实施绿化美化乡村265条，绿色生态空间不断拓展。

　　（六）全力改善和保障民生，人民群众拥有更多获得感

　　十件民生实事全面完成。完成精准扶贫农村公路硬底化742.7公里。城乡居民基础养老金每人每年增加336元。城乡居民医保各级财政补助标准每人提高到490元。解决2290条自然村120万人安全饮水和通自来水问题。建成保障房960套。建成624座村级污水处理设施。完成293条小街小巷路灯安装，实现市区小街小巷路灯全覆盖。新增市区公办义务教育学位4805个，新增学前教育学位2.26万个。人均基本公共卫生服务经费达到55元。建成村级公建民营卫生站500间。

　　社会民生保障有力。全市财政社会民生支出394.2亿元，占一般公共预算支出比重82.6%。落实民生微实事专项资金5680万元，完成民生微实事项目1840个。新增城镇就业7.9万人，失业人员再就业3.6万人。城镇登记失业率2.4%，低于全省平均水平。失业保险金调整至1089元/月，企业职工最低工资标准调整至1410元/月。建成公租房1888套。城乡居民养老保险参保率99.99%。每千名老人拥有养老床位34.8张，提前超额完成省定任务。

　　社会事业繁荣进步。加大教育支持力度，全市教育支出98.2亿元。省教育现代化先进县（市、区）达到8个。新建、改扩建幼儿园145所。高考再创佳绩。市技师学院获全国选拔赛第一，进入世界技能大赛国家集训队。三所驻湛高校入选粤东西北高校振兴计划。16家县级公立医院升级建设项目按期推进，7间中心卫生院升级建设项目主体工程基本完工。广东医科大学附属医院入选首批广东省建设全国高水平医院“登峰计划”。行政村（社区）综合性文化服务中心实现全覆盖，14个镇（街）文体广场示范点建成，41个镇（街）文化站达标升级。大型雷剧《挖宝记》入选广东反映改革开放40周年题材四大剧目。雷州石狗入选国家第一批振兴传统工艺项目；吴川粤剧南派艺术传承人林国光入选2018年国家级非遗传承人。湛江籍体育健儿在第十八届亚运会和第三届亚残会荣获一金两银三铜。民族宗教、人防、三防、海防打私、对台事务、港澳事务、档案、地方志、气象、地震、残疾人等工作取得新进步。

　　社会大局和谐稳定。“平安湛江”建设持续推进，各类警情下降18.8%，“三项目测评”在全省的排名由2017年的末位分别提升到全省第1、第9、第15；打掉黑社会性质组织3个、恶势力犯罪集团61个、一般恶势力犯罪团伙163个，得到中央督导组充分肯定。落实“四个最严”和“四有两责”要求，食品药品安全监管水平有效提升。成功防控H7N9疫情，有效应对登革热疫情。有效应对4次台风袭击，保持连续10年不发生重特大安全生产事故记录。持续开展社会矛盾纠纷排查化解工作，在全省信访工作第一轮考核中排名第5。

　　（七）全力抓好自身建设，努力打造政府新形象

　　坚持以习近平新时代中国特色社会主义思想武装头脑、指导实践、推动工作。扎实开展“不忘初心、牢记使命”主题教育，深入开展“大学习、深调研、真落实”工作。严格落实中央八项规定精神，全市三公经费和会议费支出减少；支持纪检监察机关执纪执法，落实中央、省委巡视整改取得阶段性成效。“数字政府”改革全面提速，实现镇（街）村（居）服务事项全覆盖，排名全省第一。开通“数字湛江”专栏，开发上线“粤省事·湛江”掌上政务服务，社保、公积金等162个高频公共服务事项实现一键办理。推进审批流程优化再造，探索“套餐式”并联审批，推动跨部门、跨环节的联审联办。1052个事项实现“最多跑一次”审批服务，覆盖率94%。制定政府规章2部，出具法律意见函510份。主动公开政务信息3.3万条，市政府门户网站访问量1280万人次。办理人大建议115件、政协提案304件，办复率100%。依法行政考评获省良好等次。

　　各位代表！过去一年，全市人民奋发有为，砥砺前行，书写了新的美丽篇章。这一年，习近平新时代中国特色社会主义思想深入人心、落地生根，武装了头脑，指引了方向。全市上下深学笃用习近平新时代中国特色社会主义思想，全面贯彻党的十九大精神，深入贯彻习近平总书记对广东重要讲话精神，内化于心，外化于行。这一年，市委科学决策、有力领导，凝聚了共识，统揽了全局。着眼国家和全省发展大局，市委组织实施“四大抓手”和“五大产业发展计划”，谋划建设“一通道、一港区、一示范”，符合习近平新时代中国特色社会主义思想和党的十九大精神，契合湛江实际，有效推动湛江加快建设省域副中心城市、打造现代化沿海经济带重要发展极。这一年，全市上下精诚团结、社会各界鼎力支持，凝聚了合力，支撑了发展。市委坚强领导，上下同心协力、密切配合，各界人士发挥聪明才智、贡献了智慧和力量。在此，我谨代表市人民政府，向全市人民，向人大代表、政协委员，向各民主党派、工商联、人民团体及社会各界人士，向中央、省驻湛单位，向驻湛人民解放军、武警官兵，向公安政法干警，向所有关心支持湛江发展的老领导、老同志和港澳台同胞、国际友人，表示衷心的感谢和崇高的敬意！

　　各位代表！回顾一年工作，我们也清醒地认识到，对标人民对美好生活的期盼，对标建设省域副中心城市、打造现代化沿海经济带重要发展极目标的要求，我市仍有许多问题，必须进一步加大力度解决。一是高质量发展步伐有待加快。经济体量不大，综合实力不强。创新动能不足、氛围不浓，制造业数字化、网络化、智能化程度不高。全市研发投入占GDP的比例偏低，全市高新企业数量偏少。上市公司屈指可数。污染防治任务艰巨，生态文明建设有待加强。民生欠账较多，在全省扶贫任务最重，群众关心的教育、医疗、住房、养老等问题尚未很好解决。维护社会稳定压力仍然较大。二是发展的平衡性和协调性有待提升。地方财力、发展速度与省域副中心城市、现代化沿海经济带重要发展极地位不匹配。尤其是县域经济薄弱，无一进入“中国百强县”。三是对外开放水平不高，营商环境有待改善。一些干部因循守旧思想严重，存在“守靠要怨”的思想，缺乏聚焦发展、主动谋划的责任感和紧迫感。一些部门和工作人员服务意识和能力不强，行政效能不高，作风不实，不作为、慢作为、乱作为现象依然存在。我们将以问题为导向，主动担当，以富有斗争的精神，战胜困难、掌握主动、赢得未来！

　　2019年工作安排

　　2019年，是新中国成立70周年，是决胜全面建成小康社会第一个百年奋斗目标的关键之年，也是落实习近平总书记对广东重要讲话精神的开局之年，做好今年工作至关重要。

　　当前，我市正迎来加快建设省域副中心城市、打造现代化沿海经济带重要发展极的重大机遇。同时，我们也面临严峻挑战。受国际多重因素影响，世界经济增长放缓。环境更加复杂，困难挑战更多，国内经济运行稳中有变、变中有忧，下行压力加大。我市较为落后的基本市情仍未根本性改变，加快发展的任务繁重艰巨。面对挑战，全市上下要化压力为动力，全力提高干的本领，增强干的担当，提升干的实效，努力把湛江发展的优势和机遇落实在具体工作上、体现在发展成果上、展现在湛江大地上！

　　2019年我市经济社会发展的总体思路：高举习近平新时代中国特色社会主义思想伟大旗帜，全面贯彻党的十九大和十九届二中、三中全会以及中央经济工作会议精神，贯彻落实习近平总书记对广东重要讲话和对广东工作一系列重要指示精神，认真落实省委十二届六次全会精神和省委、省政府“1+1+9”工作部署，以及市委十一届七次全会精神，坚持稳中求进工作总基调，坚持新发展理念，坚持推动高质量发展，坚持以供给侧结构性改革为主线，全面深化市场化改革、扩大高水平开放，聚力推进“一通道、一港区、一示范”建设，大力推进“四大抓手”、做好“四篇文章”、实施“五大产业发展计划”，进一步稳就业、稳金融、稳外贸、稳外资、稳投资、稳预期，加快建设省域副中心城市，全力打造现代化沿海经济带重要发展极，为全面建成小康社会打下决定性基础，以优异成绩向中华人民共和国成立70周年献礼。

　　2019年我市经济社会发展的预期目标：生产总值增长7%左右；规上工业增加值增长7%；固定资产投资增长12%；社会消费品零售总额增长10%左右；外贸进出口总额实现正增长；实际利用外资增长3.5%；港口吞吐量达到3.15亿吨；一般公共预算收入增长8%；居民人均可支配收入增长7%；消费价格涨幅控制在3%以内；城镇登记失业率控制在3.5%以内。

　　2019年政府工作坚持以问题、目标、成效为导向，采取“工作计划化、计划项目化、项目目标化”倒逼各项任务的落实。着重抓好以下八个方面工作。

　　（一）全面深化改革开放，加快营造一流营商环境。用好改革开放“关键一招”，以改革开放新举措力促营商环境大提升。

　　建设全面开放新格局，深度融入区域一体化发展。积极谋划对外开放新平台，为全省形成全面开放新格局作出新探索。统筹抓好“一通道、一港区、一示范”建设，积极申报国家现代化沿海经济带雷州半岛先行区。积极参与共建国际陆海贸易新通道，支持湛江港集装箱运输、海铁联运、水水中转和内陆无水港业务发展。主动融入粤港澳大湾区，加快与深圳南山合作共建高新产业园，推动湛江港和东海岛纳入广东自贸区，申报湛江深水港与粤港澳大湾区港口合作项目，推动一批项目列入粤港澳大湾区重点项目库和基础设施、产业发展等专项规划。主动融入北部湾城市群发展，深化全领域全方位合作，推进建设湛江－北海粤桂北部湾经济合作区。发挥湛江连接海南自贸区的咽喉要道作用，与海南相向而行、错位发展，在旅游开发、港口贸易等领域对接借鉴海南改革开放的新举措，主动承接海南自贸区加工贸易转移，加快琼州海峡航运资源整合，共建琼州海峡经济带，打造海南现代服务业重点延伸区和扩散地。加强与黑龙江省绥化市对口合作。全面实施外商投资准入前国民待遇加负面清单管理制度。积极支持海关基础设施建设和创新发展，推动湛江机场、湛江港徐闻港区等重点口岸扩大开放，拓展海关特殊监管区功能，申报设立综合保税区和自由贸易试验园区。启动创建湛江港“国际卫生港”。办好水博会、东盟·农博会和小家电博览会。

　　全面完成市、县两级政府机构改革。以推进政府治理体系和治理能力现代化为导向，科学设置政府机构，合理配置行政职能，切实做好“三定方案”制定、人员转隶、资产划转等工作，确保3月底前完成改革任务。统筹推进、同步实施事业单位改革，推动相关行政职能回归行政机关。深化综合行政执法改革，整合组建市场监管、生态环保、文化市场、交通、农业等五个领域综合执法队伍。

　　深化“放管服”改革。全面简政放权，继续推进强县放权，进一步压减政府部门权责清单事项数量，下放自然资源管理、市场监管等领域行政职权；推进简政强镇，整合县镇两级审批、服务、执法等方面力量和职能，理顺县镇职责关系。坚持抓大放小、放实放责、事权适配、权责一致，继续抓好行政审批委托制改革，推动市、县两级职能部门提质增效。继续深化商事制度改革，推进“照后减证”，深化“证照分离”改革，完善中介服务超市，降低制度性交易成本。落实“非禁即入”，全面实施市场准入负面清单制度。强化市场综合管理，推动“双随机、一公开”监管全覆盖，规范和维护市场秩序。全面推进信用监管，严格落实经营异常名录管理和严重违法失信企业名单管理制度。实施企业投资项目分类管理和落地便利化改革，实现开工前政府审批时间民用建筑项目不超过41个工作日、工业项目不超过45个工作日。

　　深入推进重点领域改革攻坚。坚持全面发力、纵深推进，推动省委部署的18项重大改革任务落实落细落地。完善深化营商环境综合改革行动方案，推进开发区、坡头区营商环境综合改革试点。加快“数字政府”建设，构建“管运分离”的电子建设管理新体制，推进政务信息系统整合，打通“数据孤岛”。深化财税体制改革，重点加强对财政金融等政策研究，科学划分市和县（市、区）财政事权和支出责任，财政资金全面实施绩效管理；做好社保费和非税收入划转工作。切实防范金融风险，加快农合机构改革，全面完成农信社改制组建农商行。深化国资国企改革，支持市属企业与央企、省企合作，推进市属国企改制、兼并重组。完成市图书馆、市文化馆、市博物馆法人治理结构改革。改革社会组织管理制度。

　　（二）加快构建现代化快速立体交通体系，对接全国沿海综合运输大通道。实施交通项目60项，完成投资210亿元，推动机场升级、高铁进城、高速成网、半岛成环、港口成群，加快构建现代化快速立体交通体系。

　　建设现代化国际枢纽空港。开工建设湛江国际机场主体工程和机场大道，年内完成总体形象进度30%以上；规划建设高铁机场站，推进空铁公路“零换乘”，完成空港经济区规划并启动征地拆迁前期工作，努力建成全国一流、辐射国内和东南亚地区的综合国际性干线机场及配套能力。推进高铁建设。开工建设广湛高铁、合湛高铁、湛海高铁，加快张海高铁前期工作，构建以湛江为中心、2小时连接粤港澳大湾区、北部湾城市群、海南自贸区的放射状高铁大通道。完善沿海高快速路网体系。打造“三环八支”高快速路网络体系。规划建设广东滨海旅游公路湛江段，谋划建设东头山岛跨海大桥。开工建设疏港大道扩建、西城快线二期、茂湛高速改扩建工程和环城高速南三岛大桥。加快建设东雷高速、汕湛高速吴川支线、调顺跨海大桥。推动玉湛高速完成总体形象进度90%。加大地方财政配套力度，加快国省道升级改造，年度完成投资50亿元。推进港口建设。开工建设湛江港40万吨级航道、宝钢湛江钢铁3号高炉配套码头、湛江港拆装箱服务区一期工程。规划建设巴斯夫项目配套码头、雷州港区乌石作业区通用码头。加快东海岛港区杂货码头、霞山港区通用码头建设。建成中科炼化一体化项目配套码头一期。建成10项重点输变电项目。谋划建设东海岛铁路东海岛港区支线、宝满港区支线和粤海铁路乌石支线，全面提升港口功能与国际化水平。落实与招商局集团“1＋N”战略合作，加快建设湛江国际邮轮港综合体，深化与周边港口合作，拓展内陆无水港。基本完成徐闻港南山客货滚装码头和综合交通枢纽建设，确保年内开港，打造对接海南自贸区的黄金通道。

　　（三）着力构建现代化产业体系，强化现代化沿海经济带重要发展极产业支撑。坚持供给侧结构性改革主线不动摇，在“巩固、增强、提升、畅通”上下功夫，引进龙头大项目，打造优质大集群，以“五大产业发展计划”为抓手，切实做好“四篇文章”，实施重点项目242个，计划投资460亿元、增长10%，强化省域副中心城市、现代化沿海经济带重要发展极的产业支撑。

　　加快打造世界级绿色高端临港产业集群。建立重点项目工作台账，强化时间倒排、任务倒逼，以重点项目为依托，建设具有国际影响力的高新产业集群、东海岛重化产业循环经济示范基地和海洋产业集聚区。推进重大工业产业项目达产增效，实施重大临港产业项目17项，完成投资177.1亿元，做强做优钢铁产业链，拓展延伸石化、造纸产业链。举全市之力服务巴斯夫精细化工一体化基地项目落地，动工建设首期装置项目；谋划建设世界一流的国际化工产业园区。建成中科炼化项目、大唐国际雷州电厂、湛江——廉江成品油管道和输气管道配套项目。支持乌石17-2油田群开发项目动工建设，开展大型设备采办。培育壮大海上风电产业链，加快广东粤电湛江外罗海上项目建设，实现年内30台风机并网。

　　力促项目招大引强选优。抓住国家对新上重大项目能耗指标实行单列的机遇，大力实施精准招商，建立重大项目洽谈引进“绿色通道”，吸引更多优质产业项目落户我市。强化督办考核。建立招商工作考核机制，对照招商签约项目清单，压实县（市、区）主体责任，推动签约项目开工率达70%以上。强化要素保障。优化重点项目审批“直通车”服务，实施投资20亿元以上重大外资和重大制造业项目用地“应保尽保”，加大批而未供、供而未用土地整治力度。强化项目谋划。加强项目储备，动态更新三年滚动投资计划。做实策划论证、项目申报、筛选储备，推动更多项目列入国家、省重点项目长期规划和年度计划。

　　贴心服务振兴实体经济。大力推动民营经济发展。落实省“实体经济十条”“民营经济十条”政策，出台促进民营经济高质量发展和小微工业企业上规模的实施意见，建立中小企业（民营经济）发展机制，继续开展暖企“三服务”活动，落实市领导挂点服务重点民营企业制度，进一步优化环境、降低成本、扶持创新，支持民营经济壮大做强。开展民营工业经济奖励评定和“一企一策”“一事一议”精准帮扶。完善金融政策，举办中小微企业日活动和政金企投融资对接会，推广互保金贷款、政银担合作。支持中小企业利用多层次资本市场开展直接融资。加大技能培训支持力度，加强企业用工服务管理，帮助企业解决用地招工难题。发布年度工业百强与纳税百强。推动传统产业转型升级。深入实施工业企业技术改造三年行动计划，补足和提升产业链水平，加快建设小家电、羽绒两个示范性产业公共服务平台，建设省小家电智能制造区域创新中心。大力推动积极的财政政策，支持家电、食品制造等200家企业技改，支持企业推进国家和省智能制造14个示范试点，支持企业建设省级企业技术中心25个。

　　推动现代服务业提速发展。推动先进制造业和现代服务业深度融合，改善消费环境，完善金融服务政策，多渠道扩大内需。发展数字经济。加快建设全光网城市，推动“千兆光纤进小区、百兆光纤进家庭”，提升城乡4G基站覆盖能力。大力发展工业互联网、云计算等现代信息服务业，建设工业互联网创新应用试点示范区，推动企业“上云上平台”，支持湛江高新区建设“互联网+”小镇。做好滨海旅游文章。扎实推进广东省全域旅游示范市创建工作。加快将特呈岛、吉兆湾打造成为高端滨海旅游休闲度假区。认真做好华侨城集团全域旅游合作开发等重大项目前期工作，力争华侨城集团欢乐海岸、大湖光岩旅游景区、雷州骑楼古街旅游区等项目今年动工建设。加强与海南的旅游合作，共同打造琼州海峡热带旅游目的地。打造高品质旅游项目，推动旅游与文化、商贸等产业深度融合发展，走内涵式发展道路。提升传统服务业。推进电子商务发展，发展健康养老等生活性服务业。实施商圈消费引领工程，做大商圈规模，打造高端消费新高地。

　　着力提升外贸抗风险能力。出台贯彻落实省“外资十条”实施意见，做好政策协同。推动“单一窗口”标准版口岸和应用项目全覆盖，推进货物进出口通关整体时间再压缩1/3。依托湛江保税物流中心（B型）拓展网购保税进口、保税展示交易等业务，推进申建湛江综合保税区。拓展境外经贸合作网络。扩大进出口贸易，削减进口环节制度性成本，提升进口规模和质量，推动出口市场多元化。

　　（四）大力实施创新驱动发展战略，强化高质量发展新动能

　　着力建设国家高新区和海洋经济发展示范区。按照“一区多园”模式完善管理运行机制，推动高新区高质量发展。支持开发区创新临港钢铁石化循环经济发展模式，完成钢铁配套园区3条配套道路及排污管网建设，推动钢铁配套园区中冶宝钢等20个项目建成投产。加快推进国家高新区海东园区规划建设，抓紧启动广东省实验室项目建设。加快海洋科技产业创新中心建设，启动建设中关村湛江科技文创园。深入推进蓝色海洋综合开发计划，着力建设全国海洋经济创新发展示范城市。完成湛江市军民融合发展战略规划编制，加快推进国家级军民融合示范区建设，规划建设军民融合产业保障基地，加快中船重工合作项目落地；推动广东军民融合发展中心全面投入使用，发展军民融合船舶、舰载执法装备及深海装备、深海智能养殖产业。建设现代高科技水产种苗园区，支持水产企业“走出去”。积极引进海洋经济大型企业落户湛江。

　　营造良好创新生态。深入实施高新技术产业培育计划，大力培育生物医药、新材料等高新技术产业，力争高新技术企业总量突破230家。落实人才引进政策，加大人才引进力度。实施创新创业团队引育计划，支持企业建设院士、博士、科技特派员工作站等人才平台，推动创新创业资源集聚。出台促进科技成果转化实施办法，建设科技成果转化交易平台。强化专利质量提升工作，培育高价值专利；执行最严格的专利保护制度。

　　（五）落实区域发展新战略，提高发展的平衡性和协调性。认真落实“一核一带一区”功能区发展战略，努力推进平衡、协调发展。

　　全面提升城市发展规划。强化规划龙头引领作用，做到发展规划、城乡规划、土地利用规划、生态环境保护等“多规合一”。开展城市总体设计和调顺岛、海东高新园区等重点城市景观设计，编制高铁西站周边规划，塑造城市地域特色和时代风貌。

　　做优做强中心城区组团。推进主城区强芯提质。整合资源，强化赤坎、霞山、开发区中心城区功能，大力发展总部经济。高标准开发“旧大天然—金沙湾—调顺岛”片区。加快建设“南海之芯”特色小镇。加快推进湛江大道建设。完成民和路等5条“断头路”的征地拆迁工作。拓展城市绿色空间，建设或改造提升海田等一批休闲绿地。建成市供水一期工程，确保湛江人民喝上健康水、放心水。推进海东新区建设。按照国内一流标准启动国家高新区海东园区硬软件建设，加快中海油高温高压油气重点实验室等一批重大项目落地建设。加快省海岸带综合示范区建设，加快空港物流基地建设、南油“三旧”改造。加快海川快线扩建及军港大道海顺路建设。动工建设南调路旧城区排污工程。推进城市治理。注重在科学化、精细化、智能化上下功夫，不断提升城市管理水平。加快建成交通大数据决策平台，优化数字城管平台。加强历史文化街区、历史建筑、传统村落和南粤古驿道保护。出台城市公园管理条例，整治城市“六乱”，完成省下达的违法建设治理目标。完成瑞云中路—政通东路等9个拥堵路口综合整治，推动交通循环畅通。

　　推动县域发展壮大。推进县域新型工业化。坚持工业兴县、特色强县，支持县域产业园区完善基础设施、强化主导产业、加快扩能增效。支持佛山顺德（廉江）产业转移园建设广东省小家电智能制造区域中心。支持吴川羽绒等传统特色产业转型升级。支持徐闻、雷州承接海南自贸区政策的溢出效应，大力发展滨海旅游、清洁能源、海上设备、航运物流和仓储等产业。推进县域新型城镇化。支持遂溪与湛江市区同城化发展，加快湛江大道与遂溪大道对接。支持县（市）主城区干道改扩建，完善县（市）主城区水电燃气、通讯网络和生活垃圾、污水处理设施，推动教育、医疗等公共服务资源向县镇两级配置。

　　强力实施乡村振兴战略。深入实施乡村振兴“七大行动”，推动农业农村产业兴旺、生态宜居、乡风文明、治理有效、生活富裕，努力把短板变成“潜力板”。深化农村改革。稳步推进农村集体产权制度改革，推进村民议事协商、村民小组“五有”规范化，创建村务公开“五化”试点。全面总结推广垦造水田、拆旧复垦等改革试点经验，落实耕地占补平衡，全面探索以工补农、以城带乡机制。发展现代特色农业。培育市级示范性家庭农场、市级农民合作社示范社等新型农业经营主体300个以上。发展休闲农业、农产品电子商务、农业公园，推进农村一二三产业融合发展。推广主要粮食经济作物生产全程机械化，提升农业生产效率和设施装备水平，试点创建田园综合体。提升雷州半岛国家级现代农业示范区建设，加快省级现代农业产业园建设。实施市养殖水域滩涂规划，建设工厂化循环水养殖基地和健康养殖示范场。加强农村项目建设和资金使用管理。出台加快农村项目建设指导意见，打造“四好农村路”示范县（市、区）。完成地质灾害防治高标准“十有县”创建任务。加强农村精神文明建设。落实省“千村示范、万村整治”工程，完成全市行政村环境基础整治任务，60%以上行政村达到干净整洁村标准。推动全市文明村80%覆盖，218个省定贫困村75%达到省级文明村创建标准。

　　（六）加强生态文明建设，推动发展与美丽双赢。坚定践行习近平生态文明思想，以更大决心更大力气坚决打赢污染防治攻坚战，加快推进生态文明建设，守护好湛江碧水蓝天。

　　坚决做好污染防治工作。落实中央环保督察、海洋督察、自然资源例行督察整改，全面实施污染防治攻坚战三年行动计划，守住生态保护、永久基本农田、城镇开发边界三条控制线。推进空气质量持续向好。强化重点企业监管，加大生物质成型燃料锅炉和“散乱污”企业整治。加强柴油货车、非道路移动机械、港口船舶大气污染治理，大力推广新能源汽车，强化工地、运输扬尘和露天焚烧监管，确保空气质量保持全省前列。推进水体质量进一步改善。全面压实四级河长责任，深入推进“五清”专项行动。制定湛江市“让广东河更美”大行动实施方案，建设“万里碧道”湛江示范试点，确保地表水考核断面达标。抓紧推进劣五类水体消除、黑臭水体整治、污水管网配套建设，提标改造12座污水处理设施。新增城市（县城）污水处理能力46.5万吨/日。推进土壤和固体废物污染防治能力提升。完成重点行业关闭搬迁企业用地土壤污染状况调查，实施农用地分类管理，严格建设用地准入管理，强化土壤污染管控修复。推进垃圾分类处理，加快推动建设吴川、徐闻、雷州垃圾焚烧发电厂和遂溪危险废物综合处理中心项目。

　　提升生态文明创建水平。深化生态文明体制改革，建立健全国土空间开发保护、生态文明绩效评价考核和责任追究等制度。推进创建国家生态园林城市。深入实施“山水林田湖草”一体化生态保护和修复，继续实施新一轮绿化湛江大行动，绿化美化乡村100条，营造林5万亩，抓好热带季雨林和沿海防护林营建等林业重点生态工程，加快雷州半岛生态修复。加快近岸海域和海岸带综合整治。加强环保监管和综合执法，形成惩治环境违法行为的高压态势。

　　大力推动绿色低碳循环发展。推进“循环城市”“海绵城市”建设，采用透水铺装、下沉式绿地、植草沟等形式建设水系湿地、绿地广场、城市道路。推进土地出让与环保投资、税收就业相结合。鼓励工业领域节能技改、清洁生产，发展绿色建筑，鼓励发展装配式建筑。倡导绿色生活生产方式，持续推进“巩卫”“创文”工作，积极创建绿色学校、绿色社区，构建环保共治体系。

　　（七）加快保障和改善民生，确保发展成果全民共享。坚持以人民为中心的发展思想，紧贴人民群众对美好生活的向往，加大优质民生供给，让群众的获得感、幸福感、安全感更加充实、更有保障。

　　办好十件民生实事。新增市区义务教育学位1万个、全市学前教育学位1.2万个。城镇、农村低保对象最低生活保障人均补差水平分别从每月503元、228元提至554元、251元；特困人员基本生活标准按不低于当地最低生活保障标准的1.6倍、且不低于当地现行特困人员基本生活标准确定。城乡居民养老保险基础养老金从每人每月148元调整至170元。人均基本公共卫生服务经费增至60元，建成5县（市）急救医疗指挥中心。完成霞山水质净化厂扩容提质工程，动工建设赤坎水质净化厂扩容提质工程。整治41座镇级垃圾填埋场。建设农村公路安全生命防护工程570公里，完成乡道改造100公里、村道路面硬底化500公里。按国家二级馆以上标准升级改造市文化馆。新建、升级改造42座城市公厕。推进人民大道交通综合治理。

　　坚决打赢脱贫攻坚战。开展“千企帮千村、脱贫奔小康”精准帮扶。深入实施产业扶贫、就业扶贫，强化扶贫资金使用，推广生产奖补、以工代赈，实施贫困户信用等级评定及奖补，建设一批带动能力强、贫困人口参与度高、经济效益好的扶贫产业。加强与柳州扶贫协作。

　　强化民生保障。促进高校毕业生等重点人群多渠道就业，新增城镇就业7万人、失业人员再就业3万人。深入推进住房公积金政策普惠，完善住房市场体系，构建住房保障体系。新开工公租房1200套，发放住房货币补贴230户。提升医疗救助比例，把更多救命救急的好药纳入医保。推动全市每千名老人拥有床位36张以上，残疾人接受基本康复服务比例达70%以上，生活不能自理特困人员集中供养率达30%，让困难群众生活更有尊严。做好退役军人服务保障工作。深化双拥共建，大力支持驻湛部队改革发展，巩固军政军民团结。加强动物防疫防控，确保群众吃上放心肉。全力办好民生微实事，努力解决好群众身边的小事、急事、难事。

　　优先发展教育事业。增加教育投入，加快创建广东省推进教育现代化先进市，办好人民满意的教育。实施新一轮“五年行动计划”，大力建设公办、普惠性民办幼儿园和城区义务教育学校，支持发展高质量民办教育。办好农村小规模学校和乡镇寄宿制学校。落实第二期特殊教育提升计划。支持广东海洋大学等高校建设广东省重点建设学科，建成广东海洋大学寸金学院新校区二期。加快建设广东医科大学、岭南师范学院、湛江一中新校区。深化教育综合改革。锻造高素质专业化创新型教师队伍，合理核定教职工编制，优化中小学教师资源配置，落实中小学教师平均工资收入“两个不低于”和农村教师生活补助，抓好农村教师免费轮训。

　　大力推进“健康湛江”建设。全力推进市妇幼保健院新院建设、市公共卫生医院新建、市第二中医医院改扩建项目；加快推动市第一中医医院搬迁改造项目。提升基层医疗卫生服务水平，加快推进16家县级公立医院升级建设，完成7间镇级中心卫生院升级改造。加快发展“互联网+医疗健康”，建设市级区域全民健康信息综合管理平台。深化公立医院综合改革，加强中医药服务能力建设，努力创建“国家中医药综合改革试验区”。建立健全覆盖全民、城乡统筹的多层次医疗保障体系，推进医疗、医保、医药“三医联动”改革。加强对民营医院的监管，从严打击骗保行为。

　　繁荣发展文化体育事业。实施“传统工艺振兴工程”，建设一批非遗工作站。推进县级“三馆一站”公共文化服务设施开放改造。启动建设湛江“公共文化云”。重视红色教育基地雷州青年运河展览馆建设，开展文化下乡，办好“红色文艺轻骑兵”等文化品牌活动。倡导全民健身，打造环雷州半岛200公里骑行等群众体育品牌赛事。积极备战第二届全国青年运动会。支持市足协建设10个振兴乡村足球小镇示范点。

　　打造共建共治共享社会治理格局。深入开展扫黑除恶专项斗争，组织开展“平川2019”“飓风2019”等各类打击违法犯罪专项行动，强力推进全民禁毒工程。推进“雪亮工程”建设，打造“智慧新警务”。推进粤西警务训练基地建设。推进信访工作改革。落实党政同责，强化应急管理、食品药品安全监管。做好妇女儿童、民族宗教、外事侨务、海防打私、三防、人防、档案、气象、地震等工作。

　　（八）切实加强自身建设，建设人民满意的阳光法治服务型政府。紧贴新任务新要求，全面加强政府自身建设，深入推进政府职能转变，为人民提供优质高效服务。

　　加强政府系统党的领导和党的建设。以政治建设为统领，提高政治站位，坚持把党的政治建设贯穿政府工作各领域、全过程。认真开展“不忘初心、牢记使命”主题教育，深入开展“大学习、深调研、真落实”工作，牢固树立“四个意识”，坚定“四个自信”，始终做到“两个维护”。严明政治纪律和政治规矩，落实新形势下党内政治生活若干准则，涵养政府系统风清气正的政治生态。强化思想建设、作风建设、纪律建设，落实中央省委巡视整改，确保党中央国务院、省委省政府和市委决策部署落地生效。

　　建设法治政府、廉洁政府。加强法治建设，出台完善行政决策程序系列制度，强化制度引领，促进依法行政。严格遵循行政决策程序；落实政府法律顾问制度。借力高水平智库，运用大数据分析，强化高质量决策的智力支撑。依法接受人大法律监督、工作监督，自觉接受政协民主监督。坚持严格规范公正文明执法，深化政务公开，让权力公开透明、阳光运行。严格落实中央八项规定精神，整治各种隐形变异“四风”问题。严格履行“一岗双责”，从严控制“三公”经费和一般性支出。主动接受党内监督、监察监督，积极配合党委巡视巡察，全力支持纪检监察机关执纪执法。规范政商交往行为，加快构建亲清新型政商关系，做到亲而有度、清而有为。

　　建设人民满意的高效政府。以机构改革为契机，科学配置行政资源。加快“数字政府”建设，推动更多事项“掌上办”“指尖办”。深化12345市民服务热线、网上问政等平台建设，加大涉企服务力度，打通服务群众“最后一公里”。推动政府工作项目化管理，落实“四个全面”绩效考核，发挥“两函两书”督查利剑作用，发挥领导干部“头雁”效应，激励广大干部“在状态、有激情、敢担当、出实效”，引领全市人民焕发干事创业精气神。

　　各位代表！使命呼唤担当，奋斗赢得未来。让我们更加紧密地团结在以习近平同志为核心的党中央周围，高举习近平新时代中国特色社会主义思想伟大旗帜，在市委坚强领导下，不忘初心、牢记使命，只争朝夕、夙夜在公，加快建设省域副中心城市，打造现代化沿海经济带重要发展极，以优异成绩向中华人民共和国成立70周年献礼！
